# Bilateral gestational gigantomastia

**DOI:** 10.11604/pamj.2021.39.16.29374

**Published:** 2021-05-06

**Authors:** Saad Benali, Jaouad Kouach

**Affiliations:** 1 Department of Gynecology and Obstetrics, Military Hospital of Instructions Mohamed V of Rabat, Rabat, Morocco,; 2 Department of Gynecology and Obstetrics, University of Medicine, Souissi, Military Hospital of Instructions Mohamed V of Rabat, Rabat, Morocco

**Keywords:** Gestational gigantomastia, macromastia, pregnancy

## Image in medicine

Gestational gigantomastia is a rare disease characterized by diffuse, extreme, and incapacitating enlargement of one or both breasts during pregnancy. Although benign, it can lead to a great social, emotional, and physical disability. A good and complete knowledge regarding this rare but distressing clinical situation is a must among all practicing physicians especially obstetricians. We report the case of a 30-year-old woman, admitted for a bilateral massive hypertrophy of the breast occurring on pregnancy and with progressive evolution. She had four pregnancies and two born-infants. Biological exams have shown a hyperprolactinemia. Pathological exam of the mammary biopsy had shown a benign hyperplasia. Medical treatment of our patient by Bromocriptin was inefficient. She has had a bilateral mastectomy. Physiological enlargement of the breasts occurs at puberty and during pregnancy. It is known as gestational gigantomastia when enlargement in pregnancy becomes excessive, uncomfortable and embarrassing. Gestational gigantomastia may have far reaching effects for the mother and fetus. This rare condition is associated with considerable morbidity but may be associated with good fetal outcome. Multidisciplinary team effort in the form of obstetrician, plastic surgeon and anesthetist, and pediatrician is required for a successful fetomaternal outcome.

**Figure 1:**
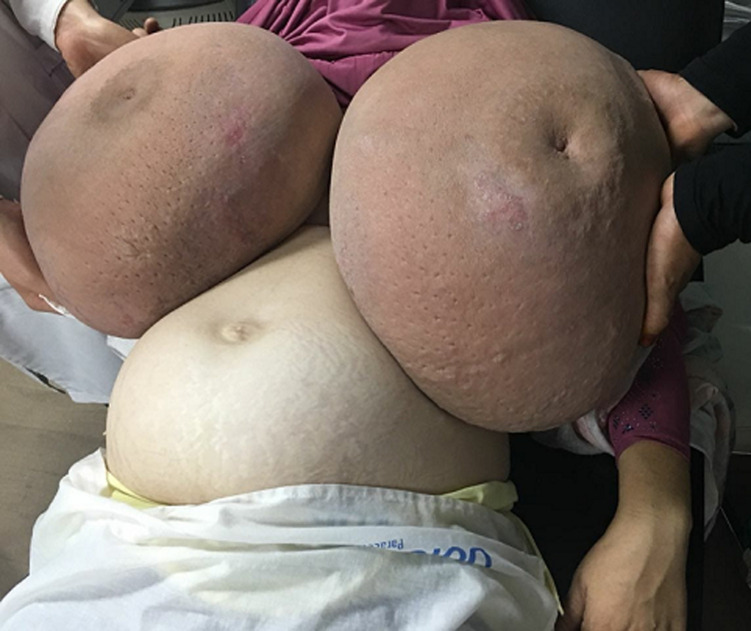
bilateral gestational gigantomastia

